# Revisiting the big five–academic performance association: a one-stage meta-analytic structural equation modeling reanalysis of 84 studies

**DOI:** 10.3389/fpsyg.2026.1769823

**Published:** 2026-03-11

**Authors:** Gul-E-Zahra, Junhua Dang, Yuhao Cui, Jie Liu, Meng Qi, Weiling Wang

**Affiliations:** 1 Institute of Social Psychology, School of Humanities and Social Sciences, Xi’an Jiaotong University, Xi’an, China; 2 Department of Surgical Sciences, Uppsala University, Uppsala, Sweden

**Keywords:** academic performance, big five personality traits, meta-analytic structural equation modeling (MASEM), personality-achievement relations, university students

## Abstract

Previous meta-analyses have consistently identified Conscientiousness as a robust predictor of academic performance, while findings for the other Big Five traits have been mixed or inconclusive. However, most existing meta-analytic evidence is based on zero-order correlations and does not account for the substantial intercorrelations among personality traits. Using a one-stage meta-analytic structural equation modeling (MASEM) approach, the present study reanalyzes data from 84 studies of university students (total *N* = 45,477) compiled in a previous meta-analysis to examine the unique associations between the Big Five traits and academic performance while explicitly modeling their shared variance. Conscientiousness remained the strongest predictor (*β* = 0.199, *p* < 0.001). Extraversion showed a significant negative association (*β* = −0.062, *p* < 0.001), whereas Agreeableness (*β* = 0.034, *p* = 0.046) and Openness (*β* = 0.060, *p* < 0.001) showed small positive associations. Neuroticism was not significant (*β* = −0.006, *p* = 0.771). Overall, the pattern is broadly consistent with prior meta-analytic evidence, but the structural model reveals a unique negative association for Extraversion that is not evident in zero-order correlations. This highlights the value of modeling the Big Five jointly when drawing inferences about personality–achievement relations.

## 1 Introduction

Academic achievement has long been a central outcome in educational and personality psychology. In this study, academic achievement (academic performance) refers to overall academic attainment operationalized primarily by GPA or closely equivalent objective indicators (e.g., course grades, standardized examination scores). Beyond cognitive ability, stable personality traits are widely recognized as important predictors of how students engage with academic tasks, regulate their behavior, and persist in the face of challenges. Personality shapes how students approach academic demands; for example, how they plan and monitor their learning, persist when tasks become difficult, and manage negative emotions during evaluation (Roberts and Mroczek, 2008). Consistent with this view, empirical studies show that personality is reliably associated with students’ self-regulated learning behaviors, academic motivation, and engagement, which are in turn linked to academic performance (Komarraju et al., 2011; O’Connor and Paunonen, 2007; Poropat, 2009); thus, the Big Five traits may partly relate to achievement via learning routines and coping strategies.

Among available personality frameworks, the Big Five model (Openness to Experience, Conscientiousness, Extraversion, Agreeableness, and Neuroticism), has become the dominant taxonomy in research (Costa and McCrae, 1992; John et al., 2008). In recent years, the Big Five framework has been widely examined in educational research, with growing attention to its associations with academic outcomes across contexts (Lechner et al., 2021; Mammadov, 2022; Soto, 2021; Zhang et al., 2023). Chen et al. (2025) conducted a recent meta-analysis examining the associations between the Big Five personality traits and academic achievement among university students, synthesizing evidence from 84 studies. They reported positive correlations for Conscientiousness, Agreeableness, and Openness, whereas Extraversion and Neuroticism showed near-zero associations. These findings are broadly consistent with earlier meta-analytic results and have often been interpreted as suggesting that, beyond Conscientiousness, the contribution of personality traits to academic achievement is relatively limited. This interpretation rests on an important methodological assumption that has rarely been questioned: namely, that the zero-order correlations between each Big Five trait and academic achievement can be interpreted as independent effects.

However, this assumption is problematic. The Big Five traits are not orthogonal; they are moderately inter correlated both within and across cultures. As a result, a zero-order correlation between a given trait and academic performance reflects not only the trait’s unique contribution, but also variance shared with other traits. When personality traits are examined one at a time, as is typical in conventional meta-analyses, it becomes impossible to determine whether an observed association reflects a genuine independent effect or a byproduct of correlated traits.

This issue is not merely technical. It has direct implications for theory. For example, positive correlations between Openness and academic achievement may reflect Openness’ overlap with Conscientiousness rather than a unique contribution of intellectual curiosity. Likewise, null effects for some traits, particularly Extraversion, may conceal unique associations that only emerge once other traits are controlled. Without modeling the joint structure of personality traits, conclusions about which traits truly matter for academic success remain ambiguous. To address this limitation, we applied meta-analytic structural equation modeling (MASEM) to estimate unique trait–achievement associations while explicitly accounting for shared variance among the Big Five traits.

Meta-Analytic Structural Equation Modeling (MASEM) offers a principled solution to this problem. By integrating meta-analysis with structural equation modeling, MASEM allows researchers to estimate simultaneous path coefficients from multiple correlated predictors to an outcome variable. We chose one-stage MASEM because it estimates the pooled correlation structure and structural path coefficients simultaneously within a single model, which can improve statistical efficiency and reduce potential bias introduced by separating pooling and model-fitting steps. This approach is particularly suitable when studies contribute different subsets of correlations and when the goal is to obtain joint, controlled associations among correlated predictors (Jak et al., 2021; Cheung, 2023). Moreover, one-stage MASEM provides a unified framework for moderator analyses, allowing us to model both categorical moderators (e.g., year-of-study group) and continuous moderators (e.g., cultural individualism) within the same estimation procedure when examining whether structural paths vary across study contexts. In doing so, it separates unique effects from shared variance and provides a structural representation of personality–achievement relations that is closer to theoretical assumptions about how personality operates. Building on this modeling framework, we further examined whether the unique trait–achievement associations vary systematically across study contexts.

The present study applies the one-stage MASEM approach (Jak et al., 2021) to reanalyze the same dataset of 84 studies compiled by Chen et al. (2025). Using identical primary studies but a different analytic framework, we address three questions: (1) Which Big Five traits uniquely predict academic achievement when their intercorrelations are explicitly modeled? (2) Do these structural relations differ from conclusions based on traditional meta-analytic correlations? (3) Are these relations moderated by relevant factors such as cultural individualism?

## 2 Reanalysis

### 2.1 Variable definition and measurement

The present study focuses on six key variables: the five Big Five personality traits, namely Extraversion, Agreeableness, Conscientiousness, Neuroticism, and Openness to Experience, and academic performance. Following the Five-Factor Model (Costa and McCrae, 1992; John et al., 2008), Extraversion reflects sociability, positive affect, and activity/energy; Agreeableness reflects compassion, cooperativeness, and a tendency to maintain interpersonal harmony; Conscientiousness reflects self-discipline, organization, persistence, and goal-directed behavior; Neuroticism reflects emotional instability and proneness to negative affect such as anxiety and worry; and Openness to Experience reflects intellectual curiosity, imagination, and preference for novelty and complexity. Academic performance is defined as students’ overall academic achievement, as operationalized in the original studies, most commonly measured by grade point average (GPA) or closely equivalent objective academic indicators (e.g., course grades or standardized examination scores).

Because the present study constitutes a one-stage meta-analytic structural equation modeling (MASEM) reanalysis, no new data were collected. All observed correlations were extracted from the 84 primary studies synthesized in the meta-analysis by Chen et al. (2025). We did not conduct an additional literature search and relied on the same primary-study pool identified by Chen et al. (2025). We adopted the inclusion/exclusion criteria from Chen et al. (2025). Specifically, studies were included if they sampled university/college students and reported associations between at least one Big Five trait and an objective academic performance indicator (primarily GPA). Studies were excluded if they (a) involved non-tertiary student populations, (b) did not report quantitative associations between the Big Five traits and academic performance, (c) relied on non-validated or *ad hoc* personality measures as coded in Chen et al. (2025), or (d) reported overlapping samples already included in the meta-analytic pool. For the present MASEM reanalysis, studies were required to provide at least one eligible zero-order correlation between a Big Five trait and academic performance; studies without sufficient information to derive correlations were excluded. Across these studies, Big Five personality traits were assessed using well-established and validated self-report instruments, including the NEO Personality Inventory (NEO-PI/NEO-FFI), the Big Five Inventory (BFI), IPIP-based measures, and closely related Big Five scales. Academic performance was assessed using objective achievement indicators reported in each study.

### 2.2 Analytic approach

The present study adopts the one-stage MASEM approach to reanalyze the association between the Big Five personality traits and academic performance. One-stage MASEM integrates meta-analysis and structural equation modeling within a single estimation framework, allowing correlation matrices from multiple studies to be pooled and structural parameters to be estimated simultaneously (Cheung, 2023; Jak et al., 2021). By specifying a structural model in which all predictors are entered simultaneously, this approach estimates unique path coefficients while explicitly accounting for intercorrelations among the Big Five traits. Compared with traditional correlation-based meta-analyses, one-stage MASEM therefore separates trait-specific effects from variance shared among predictors.

The analysis proceeded in three main steps. First, pairwise correlations among the Big Five traits and between each trait and academic performance were extracted from the primary studies included in the meta-analysis by Chen et al. (2025). When multiple correlations were reported within a study, we implemented a pre-specified decision rule to ensure statistical independence at the participant-sample level. Specifically, if a single participant sample contributed multiple correlations for the same construct pair (e.g., multiple academic indicators or multiple Big Five measures), we retained one eligible effect size per construct pair by prioritizing (a) overall GPA (or the closest objective aggregate indicator) over course-specific grades, and (b) the most commonly used/validated Big Five instrument when multiple measures were available. When studies reported results for distinct subsamples (e.g., different cohorts, classes, or institutions) with non-overlapping participants, these subsamples were treated as independent samples. Primary studies often reported incomplete correlation matrices (e.g., missing intercorrelations among the Big Five traits). In such cases, we retained all available correlations and relied on the one-stage MASEM estimation in webMASEM, which allows studies to contribute partially observed correlation matrices without requiring list wise deletion or *ad hoc* imputation. Studies were excluded only when they did not provide sufficient information to contribute any eligible trait–achievement correlation required for the structural model. Second, these correlation matrices were jointly synthesized and modeled within a one-stage MASEM framework, in which pooling of correlations and estimation of structural parameters occurred simultaneously. This procedure reduces bias associated with two-stage approaches and improves estimation efficiency (Jak et al., 2021).

Third, study-level moderators were examined by extending the structural model to include moderation effects on the personality–academic performance paths. The present study investigated three study-level moderators: cultural individualism (Hofstede, 2001), year of study in college (0 = freshmen and sophomores; 1 = juniors and seniors), and female proportion of the sample. Following Chen et al. (2025), we retained these moderators to maintain comparability with the original dataset and because they were available for a substantial proportion of samples. Although academic major and personality-measure type were also coded in Chen et al. (2025), several categories contained too few studies to support stable estimation and model convergence in one-stage MASEM moderator models; therefore, we did not test these moderators in the present reanalysis. Cultural individualism was operationalized using Hofstede’s national individualism index (higher scores indicate more individualistic cultures); each sample was assigned the index value corresponding to the country in which the data were collected. Year of study was coded as 0 = freshmen and sophomores and 1 = juniors and seniors following Chen et al. (2025) to maintain comparability with the original dataset. This dichotomy also provides a feasible and interpretable early-versus-late college contrast given that detailed year information was inconsistently reported across studies. Female proportion was defined as the percentage of female participants in each sample.

**Figure 1:**
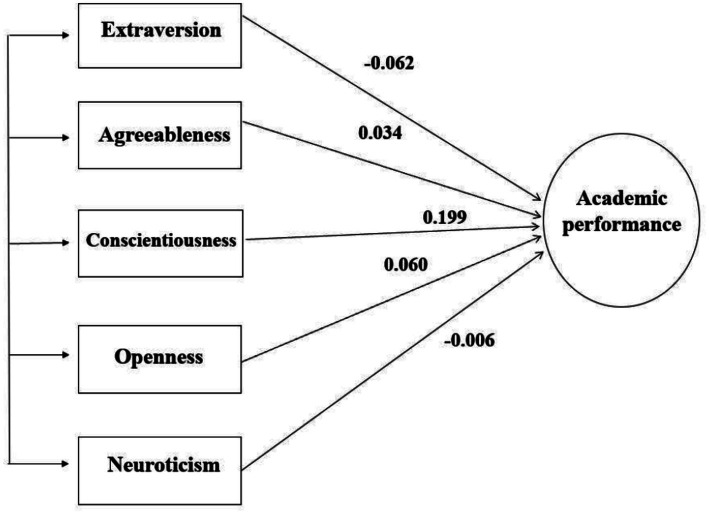
Structural model tested in the one-stage MASEM analysis.

All analyses were conducted using the webMASEM platform (Jak et al., 2021), which implements state-of-the-art one-stage MASEM procedures and provides a transparent and reproducible analytic workflow. All model specifications and estimation steps are fully determined by the reported correlation matrices from the original studies. The structural model tested in the present study is illustrated in Figure 1. The model specifies the five Big Five personality traits, which include Extraversion, Agreeableness, Conscientiousness, Openness to Experience, and Neuroticism, as correlated exogenous variables simultaneously predicting academic performance. Unidirectional arrows represent standardized structural path coefficients (*β*), reflecting unique associations between each trait and academic performance after controlling for shared variance among the Big Five traits. We report standardized coefficients because the synthesized inputs are correlation matrices and primary studies used heterogeneous measurement scales; standardized coefficients enable direct comparison of unique associations across traits within the same structural model. In contrast, unstandardized coefficients depend on the original measurement units and are therefore less comparable across studies in a correlation-based MASEM synthesis. Only direct effects are depicted; correlations among the personality traits are not shown for clarity.

### 2.3 Structural path estimates

As shown in Table 1, the one-stage MASEM results reveal a markedly different pattern from traditional correlation-based meta-analyses. Conscientiousness emerged as the strongest and most reliable predictor of academic achievement. Its standardized path coefficient remained substantial and statistically significant after controlling for all other traits, confirming that Conscientiousness exerts a genuinely independent influence on academic performance (*β* = 0.199, *p* < 0.001). This finding reinforces theoretical accounts emphasizing self-discipline, persistence, and goal-directed behavior as central mechanisms underlying academic success.

**Table 1:** Summary of the one-stage MASEM path coefficients for the associations between Big Five personality traits and academic performance.

Path to AP	*k*	*N*	One-stage MASEM		*p*
			*β*	SE	
E-AP	72	41,870	−0.062	0.017	<0.001
A-AP	72	41,714	0.034	0.017	0.046
C-AP	81	45,477	0.199	0.019	<0.001
O-AP	72	40,920	0.060	0.015	<0.001
N-AP	73	41,929	−0.006	0.019	0.771

The table presents meta-analytic estimates of the cross-sectional associations between Big Five personality traits and academic performance. Here, *k* represents the number of included samples; *N* indicates the total participant count across these samples; and *β*, SE, and *p* denote the standardized path coefficient, its standard error, and significance, respectively. E = Extraversion; A = Agreeableness; C = Conscientiousness; N = Neuroticism; O = Openness; AP = Academic Performance.

In contrast, Extraversion showed a significant negative path to academic achievement (*β* = −0.062, *p* < 0.001). This effect was not apparent in the zero-order correlations reported by Chen et al. (2025), suggesting that Extraversion’s negative association had been masked by shared variance with other traits. When these shared influences are controlled, higher Extraversion is associated with lower academic performance, consistent with accounts emphasizing distraction, social engagement, and reduced sustained attention in solitary learning contexts.

Openness to Experience displayed a small but statistically significant positive path coefficient (*β* = 0.060, *p* < 0.001). This finding suggests that intellectual curiosity and openness to new ideas may contribute modestly to academic performance once shared variance with other traits is taken into account. Agreeableness showed a weak positive association with academic achievement (*β* = 0.034, *p* = 0.046), whereas Neuroticism did not exhibit a significant unique effect (*β* = −0.006, *p* = 0.771).

### 2.4 Moderating effects

As shown in Supplementary Table S1, moderating analyses revealed one significant effect: cultural individualism significantly moderated the Agreeableness–Achievement path. Specifically, the positive association between Agreeableness and academic performance was weaker in more individualistic cultures. This pattern is theoretically consistent with the idea that cooperative and compliant tendencies are more strongly rewarded in collectivistic educational contexts. No significant moderation was observed for year of study or female proportion. Overall, the structural relations between personality traits and academic achievement appeared largely stable across these sample characteristics.

## 3 Discussion

### 3.1 Implications

The present study provides a rigorous structural reanalysis of the association between the Big Five personality traits and academic performance by applying the one-stage MASEM framework to 84 studies. By modeling all five personality traits simultaneously and explicitly accounting for their intercorrelations, this study refines existing conclusions and illustrates how analytic approach can influence theoretical interpretation of personality–achievement relations. Overall, the pattern of results is broadly consistent with prior meta-analytic evidence, while revealing important differences for specific traits, particularly for Extraversion, when shared variance among the Big Five is taken into account.

First, consistent with decades of empirical and meta-analytic evidence, Conscientiousness emerged as the strongest and most reliable predictor of academic performance (Poropat, 2009; Richardson et al., 2012). Compared with the zero-order correlations reported by Chen et al. (2025), the structural path from Conscientiousness to academic performance was stronger, underscoring that Conscientiousness exerts a genuinely unique effect rather than one inflated by shared variance with other traits. By accounting for multicollinearity among the Big Five, the one-stage MASEM approach clarifies that Conscientiousness carries explanatory power beyond what traditional correlation-based meta-analyses can reveal. This result is fully consistent with theoretical models emphasizing goal setting, sustained effort, and task engagement as the primary mechanisms linking Conscientiousness to academic outcomes (Duckworth et al., 2007).

Second, Extraversion showed a significant negative association with academic performance in the MASEM analysis, despite being near zero in the original meta-analysis. This pattern suggests a suppression effect that only becomes apparent when other traits are controlled. This finding is theoretically plausible. Extraverted students tend to allocate more time and cognitive resources to social interaction, group activities, and communication-oriented pursuits, which may reduce time available for sustained individual study. In addition, Extraversion has been linked to greater sensitivity to external stimulation and distraction (Matthews et al., 2003), which can interfere with prolonged concentration on academic tasks. The negative structural path indicates that once overlap with adaptive traits such as Conscientiousness is removed, the potential costs of extraversion for academic performance become evident.

Third, Agreeableness demonstrated a small but significant positive association with academic performance, and this effect was moderated by cultural individualism. Specifically, the association weakened in more individualistic cultures, where autonomy, competition, and personal achievement are emphasized over interpersonal harmony. In contrast, in collectivistic contexts, cooperative and compliant tendencies may align more closely with academic norms and expectations, enhancing the academic value of Agreeableness (Hofstede, 2001; Triandis, 1995).

Fourth, Openness to Experience showed a small but significant positive unique association with academic performance after controlling for the other Big Five traits. This finding suggests that intellectual curiosity, openness to new ideas, and a preference for cognitive exploration may contribute to academic success beyond their shared variance with Conscientiousness and Agreeableness. At the same time, the modest effect size indicates that the academic relevance of Openness may depend on contextual factors, such as academic discipline and the nature of achievement indicators. Because most meta-analytic studies rely on aggregated measures of academic performance (e.g., GPA) across diverse fields, domain-specific advantages of Openness, particularly in disciplines emphasizing creativity and abstract thinking, may be attenuated in overall structural models. More broadly, the unique effects of Agreeableness and Openness were modest in magnitude (*β* ≈ 0.03–0.06). Although effects of this size are often considered small, small associations can still be meaningful at the population level while remaining limited for individual-level prediction and intervention impact (Funder and Ozer, 2019). Although statistically reliable, effects of this size may have limited practical significance for individual-level prediction and should be interpreted cautiously when considering implications for educational practice. Their associations may be more detectable in context-specific outcomes (e.g., collaborative or participation-based performance indicators for Agreeableness, and learning tasks that reward exploration and intellectual engagement for Openness) than in broad, aggregated GPA composites.

Finally, Neuroticism did not exhibit a significant unique association with academic performance in the one-stage MASEM analysis. This finding is consistent with previous meta-analytic work suggesting that the academic consequences of Neuroticism are complex and may vary across contexts, levels of emotional intensity, and coping resources. This pattern may reflect competing mechanisms: emotional distress and test anxiety may undermine concentration and self-regulation, whereas evaluative concern may motivate compensatory effort in some students (Eysenck et al., 2007; von der Embse et al., 2018). Such heterogeneity could yield near-zero average effects in meta-analytic structural models. Once shared variance with other personality traits is taken into account, Neuroticism does not appear to have a consistent independent association with overall academic performance.

Taken together, these findings indicate that while many conclusions from traditional correlation-based meta-analyses remain robust, modeling the joint structure of the Big Five can yield more nuanced inferences for specific traits. In particular, the results underscore the value of structural modeling for identifying unique trait–achievement associations that may be masked in zero-order analyses. More broadly, the present study demonstrates that analytic choices are consequential for theory development, as they shape conclusions about which personality traits matter for academic performance and under what conditions.

The findings also have practical implications. Educational interventions should continue to prioritize the development of Conscientiousness-related skills, such as planning, persistence, and self-regulation, given their strong and consistent association with academic performance (e.g., structured weekly planning, assignment milestone setting, and brief progress monitoring; Theobald, 2021). For extraverted students, learning environments that provide clear structure and minimize distraction may be useful as low-cost, general study supports, particularly for tasks requiring sustained individual focus (e.g., time-blocking/Pomodoro-style study routines and scheduling social activities after study milestones). In addition, cultural context should be considered when designing personality-informed educational practices, as the academic value of interpersonal traits such as Agreeableness varies across educational systems and cultural settings (e.g., cooperative-learning or peer-support structures may be more relevant in collectivistic contexts; Kyndt et al., 2013; Hofstede, 2001). Given the modest unique effects for Agreeableness and Openness, these implications should be interpreted cautiously and framed as context-sensitive supports rather than broad trait-based prescriptions.

### 3.2 Research limitations

Despite the comprehensive scope of the present meta-analytic reanalysis, several limitations should be acknowledged. First, the analyses were based on the existing corpus of studies synthesized by Chen et al. (2025) and therefore inherit any selection processes in that corpus; as in most meta-analytic work, the results may still be susceptible to publication or availability bias. Future work should evaluate the robustness of the findings using publication-bias–sensitive approaches (e.g., sensitivity analyses or selection-model–based methods) and by increasing coverage of unpublished or hard-to-access studies where feasible. Second, academic performance was operationalized primarily through GPA or closely equivalent indices reported in the primary studies, which may not fully capture broader learning outcomes (e.g., skill acquisition, engagement, or domain-specific achievement). Future work should incorporate broader and more domain-sensitive outcomes (e.g., course-specific achievement, engagement, persistence, or skill acquisition) to examine whether trait effects differ by outcome type. Third, although the 84 studies covered multiple countries and samples, the distribution of studies across cultural and educational contexts may be uneven, potentially limiting the generalizability of the findings to underrepresented settings. Future work should expand evidence from underrepresented cultural and educational contexts to strengthen generalizability and test boundary conditions more directly. Fourth, the present one-stage MASEM estimates structural relations using study-level correlations and thus remains correlational in nature; the modeled paths should not be interpreted as causal effects. Future work should employ longitudinal and multi-wave designs (and, where possible, designs that better address confounding) to clarify temporal ordering between personality and academic outcomes. Finally, while one-stage MASEM provides a principled way to estimate unique associations among correlated predictors, the current specification focused on linear main effects and did not explicitly test potential non-linear or interactive relations among the Big Five traits. Future work should test non-linear and interaction effects among traits (and with contextual factors) to assess whether specific combinations of traits are especially beneficial or detrimental for performance.

### 3.3 Future research recommendations

Building on the limitations noted above, future research should strengthen the evidence base by (a) improving robustness checks for potential publication/availability bias, (b) broadening academic outcomes beyond aggregated GPA composites, (c) expanding coverage of underrepresented cultural and educational contexts, and (d) using longitudinal or multi-wave designs to clarify temporal ordering. In addition, future work should test more complex functional forms, including non-linear and interaction effects, and incorporate theoretically relevant covariates to better identify mechanisms and boundary conditions of personality–achievement relations.

## Data Availability

The original contributions presented in the study are included in the article/Supplementary material, further inquiries can be directed to the corresponding author.
