# Supplementary material for: Revisiting the big five–academic performance association: a one-stage meta-analytic structural equation modeling reanalysis of 84 studies
Source: Front Psychol. 2026 Mar 11;17:1769823. doi: 10.3389/fpsyg.2026.1769823 (PMC13013498; doi:10.3389/fpsyg.2026.1769823)
Supplement: Supplementary file 1 [file Supplementary_file_1.docx]

Table S1. Moderating Effects of Individualism, Year of Study, and Female Ratio on Personality-Academic Performance Relationships.

| Moderator | Cultural individualism | | | | | Year of study | | | | Female ratio | | | | | |
| --- | --- | --- | --- | --- | --- | --- | --- | --- | --- | --- | --- | --- | --- | --- | --- |
|  | k β SE *p* | | | |  | k β SE *p* | | | | |  | k | β | SE | *p* |
| E-AP | 70 | -0.01 | 0.02 | 0.421 |  | 43 | -0.05 | 0.04 | 0.258 | |  | 72 | -0.01 | 0.02 | 0.480 |
| A-AP | 70 | -0.03 | 0.02 | 0.048 |  | 43 | 0.01 | 0.05 | 0.889 | |  | 72 | -0.01 | 0.02 | 0.605 |
| C-AP | 77 | 0.01 | 0.02 | 0.461 |  | 50 | 0.011 | 0.05 | 0.826 | |  | 81 | 0.04 | 0.02 | 0.051 |
| O-AP | 70 | -0.02 | 0.01 | 0.084 |  | 44 | 0.01 | 0.04 | 0.875 | |  | 72 | 0.01 | 0.01 | 0.666 |
| N-AP | 70 | 0.02 | 0.02 | 0.241 |  | 45 | -0.02 | 0.05 | 0.646 | |  | 73 | -0.00 | 0.02 | 0.984 |

Note: k refers to the number of included samples. β represents the standardized regression coefficient, SE is the standard error, and *p* denotes the significance.

| One-Stage MASEM | | | | | |
| --- | --- | --- | --- | --- | --- |
| Correlation | k | n | r | 95%CI | τ |
| E, A | 50 | 22,710 | 0.22 | 0.16, 0.28 | 0.21 |
| E, C | 51 | 23,139 | 0.17 | 0.12, 0.22 | 0.17 |
| E, O | 48 | 22,097 | 0.25 | 0.20, 0.30 | 0.17 |
| E, N | 47 | 20,403 | -0.14 | -0.21, -0.06 | 0.26 |
| E, AP | 72 | 41,870 | -0.01 | -0.03, 0.02 | 0.10 |
| A, C | 50 | 22,710 | 0.26 | 0.22, 0.31 | 0.14 |
| A, O | 48 | 21,941 | 0.19 | 0.13, 0.24 | 0.17 |
| A, N | 49 | 22,019 | -0.16 | -0.21, -0.11 | 0.18 |
| A, AP | 72 | 41,714 | 0.09 | 0.06, 0.11 | 0.10 |
| C, O | 49 | 22,314 | 0.16 | 0.10, 0.21 | 0.18 |
| C, N | 50 | 21,432 | -0.17 | -0.23, -0.11 | 0.21 |
| C, AP | 81 | 45,477 | 0.21 | 0.18, 0.24 | 0.14 |
| O, N | 46 | 20,089 | -0.09 | -0.15, -0.03 | 0.20 |
| O, AP | 72 | 40,920 | 0.08 | 0.06, 0.11 | 0.09 |
| N, AP | 73 | 41,929 | -0.04 | -0.07, -0.01 | 0.13 |

Table S2. Meta analytic estimates from one-stage MASEM for correlations between Big Five Personality traits and Academic Performance.

Note: *p* < 0.001 for each meta-analytic correlation (r) estimate.

Abbreviations: E = Extraversion, A = Agreeableness, C = Conscientiousness, O = Openness, N = Neuroticism; AP = Academic Performance. MASEM = meta-analytic structural equation modeling.
